# Comparison of Binocular Vision Parameters Pre- and Post-EPILASIK Laser Vision Correction Surgery for Myopia in a Pilot Study – Can Vision Therapy Augment Refractive Results?

**DOI:** 10.22599/bioj.158

**Published:** 2021-01-12

**Authors:** Radhika Natarajan, Sushmitha Arcot Dandapani, Jameel Rizwana Hussaindeen

**Affiliations:** 1 Sankara Nethralaya, MRF, IN

**Keywords:** Binocular vision, accommodative amplitudes, Epilasik

## Abstract

**Purpose:**

To analyze the changes in the binocular vision parameters after bilateral Epilasik laser vision correction surgery (LVCS).

**Setting:**

Medical Research Foundation, Tamil Nadu, India.

**Study design:**

Prospective cohort study.

**Methods:**

Subjects with a best corrected visual acuity of ≤ 0.0 Log MAR scale and refractive error: < 6.00DS of myopia, < 0.75D of astigmatism, and < 1D of anisometropia were included in the study. All subjects underwent a comprehensive eye examination, LVCS workup which included corneal topography, tomography, aberrometry, and dry eye assessment prior to binocular vision assessment. Complete Binocular vision assessment which included stereopsis, fusion for distance and near, near point of convergence, phoria measurement, vergence amplitudes and facility, accommodative amplitudes, response, and facility was performed with the best corrected vision prior to LVCS, one month and six months after the surgery.

**Results:**

Twenty-five subjects of age 23.8 ± 2.9 years were included. Age ranged from 20 to 32 years. Ten were female and 15 were male. The median spherical power was –2.00DS with an inter quartile range (IQR) of –1.50DS to –3.00DS for both eyes. The median cylindrical power was plano with IQR –0.50DC to –1.00DC for both eyes. There was a statistically significant decrease in monocular and binocular accommodative amplitudes (accounting for age-related changes) as well as positive fusional vergence recovery for near between baseline and one month after surgery (p < 0.05).

**Conclusion:**

Though subjects were asymptomatic post LVCS, still there is an indication that myopic LVCS could precipitate or aggravate an existing non-strabismic binocular vision anomaly. Comprehensive binocular vision assessment and appropriate management is recommended before and after LVCS.

## INTRODUCTION

Often after Laser Vision Correction Surgery (LVCS), patients may complain of blurred vision despite a well centered treatment, good corneal healing, and negligible refractive error (Day, Powers & Faktorovich 2005). Literature postulates that poor binocular vision (BV) due to fluctuations in the ocular accommodation to be one of the reasons for blurred vision in the absence of any residual refractive error (Day, Powers & Faktorovich 2005; Prakash et al. 2007; Gots, Tassignon & Gobin 2004; Singh et al. 2015). The ocular accommodation need would increase after myopic corneal LVCS compared to spectacle correction owing to changes in the vertex distance, especially in patients with high myopia (Prakash et al. 2007). This significant increase in accommodation for near may raise concerns about asthenopia or apparent progression, especially in early presbyopia (Tsuneyoshi et al. 2014). Special care should be taken in patients who have a preoperative history of strabismus surgery, an overcorrection or under correction of refractive error in one or both eyes, anisometropia, and pre-existing binocular vision (BV) dysfunction. As LVCS could precipitate or aggravate an existing accommodation or vergence dysfunction anomaly, it is necessary to assess the BV function prior to any LVCS. As far as our knowledge, there are no literatures reporting the changes or fluctuations in BV parameters before and after LVCS in young individuals. The aim of this pilot study is to analyze and understand the changes in the BV parameters before and after LVCS in young individuals, specifically following an Epilasik procedure.

## METHODS

This prospective cohort study was carried out at the BV clinic of a tertiary eye care centre. All subjects in the study were between 20 and 32 years of age, with corrected distance visual acuity ≤ 0.0 Log MAR scale. The subjects included in the study were otherwise asymptomatic and had a refractive error: < 6.00DS of myopia, < 0.75D of astigmatism, and < 1D of anisometropia. Subjects who had constant strabismus, any history of ocular surgery, and under the medication for anti-psychotic or anti-epileptic medications or any medicines that are known to affect or influence the accommodation were excluded from the study. All consecutive subjects who were eligible for the Epilasik procedure, which was the preferred choice of treatment in low to moderate myopes, and those who fulfilled the inclusion criteria, underwent a comprehensive BV assessment. Epilasik surgery is the LVCS preferably for thin corneas and low myopia with minimum risk of post-operative ectasia.

The research was approved by the Institutional review board and Ethics Committee and adhered to the Tenets of the Declaration of Helsinki. All subjects, after obtaining an informed consent, underwent a comprehensive eye examination, LVCS workup, which included corneal topography (TMS-4, Tomey Corp), Tomography (Pentacam HR, Oculus Optikgerate GmbH), Aberrometry, and dry eye assessment prior to BV assessment. BV assessment was performed prior to LVCS, and at one- and six-month follow-ups after the surgery. Most subjects were spectacle users and assessments were made with spectacle correction.

### PARAMETERS TESTED

The BV parameters assessed included stereopsis, fusion for distance and near, near point of convergence (NPC), phoria measurement for distance and near, vergence amplitudes and facility, accommodative parameters – accommodative amplitudes, response, and facility. Sensory evaluation was performed for distance and near using Worth four dot test and Randot stereo test at 40 cm. The magnitude of heterophoria was measured using Bernell Muscle Imbalance Measure (MIM) card (subjectively) and Prism bar cover test (objectively). Near point of convergence was measured using the Astron international rule using an accommodative target using a linear target of reduced Snellen 6/9 size. Step vergence amplitudes for distance and near was assessed using prism bar for distance and near with linear targets sized one line better than the corrected distance visual acuity. Vergence facility was assessed using 12 diopters base-out/3 diopters base-in vergence flippers in cycles per minute using a linear 6/9 accommodative target as a fixation stimulus. Accommodative response was assessed using monocular estimate method (MEM) of dynamic retinoscopy over the subjective acceptance at 40cm. Near point of accommodation (NPA) was measured by push-up method using 6/9 target and the amplitude of accommodation was obtained by converting it to diopters. Monocular and binocular accommodative facility was assessed using +/–2.00 DS flipper lenses at 40cm in cycles per minute. The detailed protocol is explained in a previous publication (Dandapani, Padmanabhan & Hussaindeen 2020).

BV assessment was performed before LVCS and repeated one month and six months after the LVCS procedure. The follow-up included visual acuity and residual refractive error measurements which were taken into account for BV testing.

### STATISTICAL ANALYSIS

Statistical analysis was performed using SPSS version 20 (SPSS Inc., Chicago, Illinois, USA). As the sample size of the study was less than 30, non-parametric tests were used for the analysis. Non-parametric Wilcoxon signed-rank test was performed to compare two paired groups, and Non-parametric Friedman test was used to compare three paired groups. Post hoc tests were performed using Wilcoxon signed-rank test with adjusted Bonferroni correction. Spearman correlation analysis was used to assess the correlation between age and changes in accommodative amplitudes from pre-refractive surgery baseline to follow-up visits.

## RESULTS

Twenty-five subjects who underwent Epilasik surgery were included in the study, age ranging between 20 and 32 years (23.8 ± 2.9 years). Out of 25 subjects, 10 were female and 15 were male. The median refractive error (spherical equivalent) before surgery was –2.00DS in right eye with an interquartile range (IQR) of –2.00 DS to –4.00DS, and –3.00DS in left eye with IQR –2.00DS to –4.00DS respectively. The median spherical power was –2.00DS with IQR –1.50DS to –3.00DS for both eyes. The median cylindrical power was plano with IQR –0.50DC to –1.00DC for both eyes respectively.

### COMPARISON OF BV PARAMETERS PRE AND POST LASIK (ONE MONTH)

**Figure 1:**
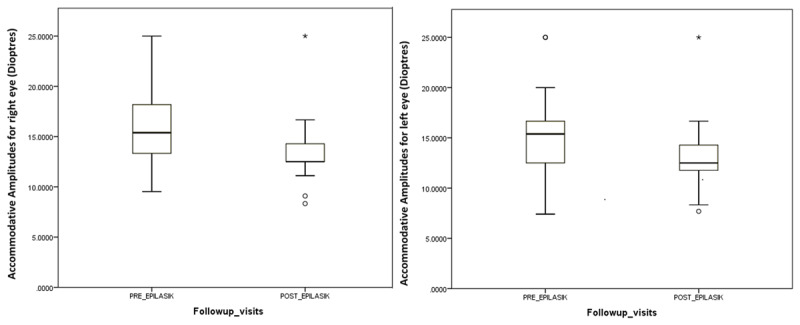
Graph shows the comparison of monocular accommodative amplitudes **using push-up test** between baseline and 1^st^ follow-up (n = 25).

The vergence and the accommodative parameters were assessed prior to refractive surgery (baseline values) and at one-month follow-up after LVCS procedure for all the patients (***Table 1***). Statistically significant decrease was noted in monocular and binocular accommodative amplitudes (***Figure 1***) and near positive fusional vergence recovery (Wilcoxon Signed rank test, p < 0.05). An average of 3 cpm (cycles per minute) reduction was noted in right eye accommodative facility for 13 subjects and in left eye accommodative facility for 14 subjects pre- and post-Lasik. But there was no statistically significant difference in the accommodative facility of two visits.

**Table 1:** Comparison of BV parameters before and one month after LVCS (n = 25). PD - Prism Dioptre, NPC - Near point of convergence, AA - Accommodative amplitudes, PFV - Positive fusional vergence, NFV - Negative fusional vergence, cpm - cycles per minute, MEM - Monocular estimation method, OD - Oculus dexter, OS - Oculus sinister, OU - Ocular uturque, IOR - Interquartile range, D - Dioptres.


PARAMETERS	BASELINE		1^ST^ FOLLOW-UP (1 MONTH)		P-VALUE
	
	MEDIAN	IQR	MEDIAN	IQR	

**Stereo-acuity (arcs sec)**	40	40–45	40	40–50	0.06

**Vergence Parameters**					

**Distance magnitude of deviation (PD)**	0	0–1	0	0–2	0.10

**Near magnitude of deviation (PD)**	0	0.2	0	0–3	0.57

**NPC (cm)**	6	4–7.5	6	4–7.5	0.61

**NFV Distance Break (PD)**	10	6–10	10	8–10	0.83

**NFV Distance Recovery (PD)**	6	4–8	8	6–8	0.34

**NFV Near Break (PD)**	12	12–16	14	12–15	0.82

**NFV Near recovery (PD)**	10	10–14	10	10–13	0.82

**PFV Distance Break (PD)**	20	13–25	20	14–25	0.22

**PFV Distance Recovery (PD)**	16	10–18	14	10–20	0.50

**PFV Near Break (PD)**	30	25–30	30	25–35	0.20

**PFV Near recovery (PD)**	20	20–25	25	20–30	0.01*

**Accommodative Parameters**					

**Vergence facility (cpm)**	12	10–14	12	10–14	0.80

**AA OD (D)**	15.4	12.9–18.2	12.5	12.1–14.3	0.038*

**AA OS (D)**	15.4	12.5–17.4	12.5	11.4–14.3	0.014*

**AA OU (D)**	16.7	14.3–20	14.3	11.1–15.4	0.049*

**MEM OD (DS)**	1	1	1	0–1	0.16

**MEM OS (DS)**	1	0–1	1	1	1.00

**Accommodative facility OD (cpm)**	9	7–10	10	8–12	0.15

**Accommodative facility OS (cpm)**	10	8–12	8	6–12	0.21

**Accommodative facility OU (cpm)**	10	8–12	10	7–12	0.97


### COMPARISON OF BV PARAMETERS AT BASELINE, AND AT ONE- AND SIX-MONTHS FOLLOW-UP

**Figure 2:**
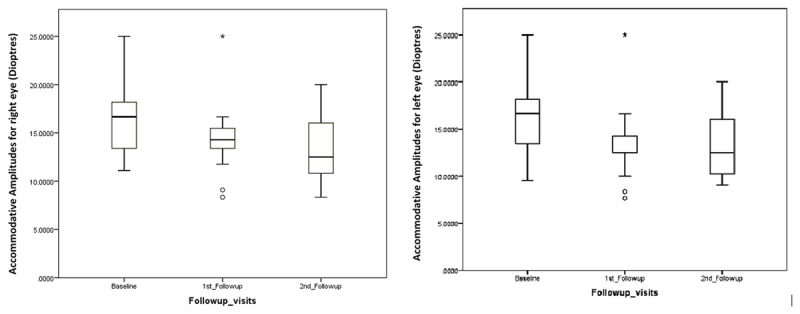
Graph shows the comparison of binocular accommodative amplitudes **using push-up test** between baseline and 1^st^ follow-up (n = 25).

At six-month follow-ups, only 15 subjects turned up for further evaluation (***Table 2***). The reduction in monocular accommodative amplitudes continued to remain statistically significant between the three follow-ups (Freidman Test, p < .05) (***Figure 2***). Post hoc analysis performed with adjusted Bonferroni showed significant difference for left eye between pre and post one-month follow-up (Wilcoxon signed rank test with adjusted Bonferroni, p = 0.008). An average of 2cpm reduction was noted in accommodative facility for three subjects in right eye and seven subjects in left eye in the 3^rd^ follow-up from baseline visit. None of the subjects had asthenopic symptoms and despite changes in accommodative amplitudes, the accommodative amplitudes remained comparable to age expected norms at all visits. There was no statistically significant difference in the phoria status, MEM, and NPC between the three visits. There was no significant correlation between age and changes in accommodative amplitudes from baseline to follow-up visits (Wilcoxon signed rank test, p > 0.05).

**Table 2:** Comparison of BV parameters before and after one month and six months after LVCS (n = 15). PD - Prism Dioptre, NPC - Near point of convergence, AA - Accommodative amplitudes, PFV - Positive fusional vergence, NFV - Negative fusional vergence, cpm - cycles per minute, MEM - Monocular estimation method, OD - Oculus dexter, OS - Oculus sinister, Ocular uturque, IOR - Interquartile range, D - Dioptres.


PARAMETERS	BASELINE		1^ST^ FOLLOW-UP		2^ND^ FOLLOW-UP		P-VALUE
		
	MEDIAN	IQR	MEDIAN	IQR	MEDIAN	IQR	

**Stereo-acuity (arcs sec)**	40	40	40	40	40	40	0.066

**Vergence Parameters**							

**NPC (cm)**	6	4–8	7	4–8	6	4–10	0.193

**NFV Distance Break (PD)**	10	6–10	10	6–10	10	8–12	0.461

**NFV Distance Recovery (PD)**	8	4–8	8	4–8	8	6–10	0.397

**NFV Near Break (PD)**	14	12–14	14	12–16	14	12–16	0.486

**NFV Near recovery (PD)**	10	10–14	12	10–14	12	10–14	0.627

**PFV Distance Break (PD)**	20	10–25	16	14–25	20	16–25	0.678

**PFV Distance Recovery (PD)**	16	8–18	12	10–16	18	12–20	0.382

**PFV Near Break (PD)**	25	25–30	30	25–35	30	25–35	0.117

**PFV Near recovery (PD)**	20	20	25	20–30	20	20–30	0.05

**Vergence facility (cpm)**	12	10–14	12	10–14	12	10–13	0.368

**Accommodative Parameters**							

**AA OD (D)**	16.7	12–18	14.3	12–16	12.5	10–16	0.04*

**AA OS (D)**	16.6	12–18	12.5	12.5–14	12.5	12–16.5	0.01*

**AA OU (D)**	16.7	15.3–20	14.3	11.1–15.3	12.5	11.1–16.7	0.05

**MEM OD (DS)**	1	0–1	1	0–1	1	0–1	0.507

**MEM OS (DS)**	1	0–1	1	0–1	1	0–1	0.582

**Accommodative facility OD (cpm)**	10	8–12	10	7–12	11	4–13	0.668

**Accommodative facility OS (cpm)**	10	8–13	10	7–12	9	2–12	0.133

**Accommodative facility OU (cpm)**	8	8–12	11	6–12	12	8–13	0.789


## DISCUSSION

In this study, we report the changes in accommodation and vergence parameters before and after Epilasik laser vision correction procedure among young individuals.

As far as our knowledge goes, there exists no literature that reports the changes in BV parameters before and after Epilasik surgery.

In our study, there was almost a 2.9 diopters reduction in monocular accommodative amplitudes at 1st month follow-up visit and 4 diopters reduction at 6th month follow-up visit. But surprisingly, none of the patients exhibited asthenopic symptoms. This could be due to the following reasons. Though there was a significant decrease in accommodative amplitudes at every visit, the range was within the normal limits based on the age expected norms (Scheiman & Wick 2008; Hussaindeen et al. 2017). The changes in accommodative amplitudes were consistent with a previous literature that also reported an increase in the Accommodative convergence/Accommodation (AC/A) ratio one-month post Laser in situ keratomileusis (LASIK), which signifies that it could cause some impact to the accommodation system post-surgery (Prakash et al. 2007). These findings are also corroborated with a decrease in accommodative facility post refractive surgery, though this was not statistically significant.

It is also interesting to note the asymmetric changes in accommodation between the right and left eyes, with a 2.4 and 4.1 diopter change in the right and left eye, for which ocular dominance might be a reason. The reason for absence of symptoms could be due to the age of the study subject itself, as all our subjects except one, who was less than 30 years of age. In our study, there was no significant difference in the accommodative response measured using MEM among the three follow-up visits. Whereas in Zheng et al. (2009) study, they reported significant differences in the accommodative response before and after SMILE surgery to different stimulus levels (P < 0.001) (Zheng et al. 2009). They concluded that the preoperative manifest refractive spherical equivalent (P = 0.006) and preoperative accommodative lag (P = 0.04) had a significant impact on postoperative accommodative lag.

It is hypothesized that the removal of prismatic effect produced by the concave lenses can make it difficult for the emmetropic eyes to converge (Prakash et al. 2007). But in our study, we did not observe any significant change in the near point of convergence or convergence amplitudes. The presence of a sustaining reduction in the accommodation present even at six-month follow-up visits emphasizes the need for long term follow-up following refractive surgery. It is possible that asthenopic systems could occur with prolonged near work or use of gadgets like laptop/mobile phones or tablets and this should be explained to the patients. Though statistically significant difference is present for some parameters, such as the near PFV recovery, other vergence and accommodative parameters should also be taken into consideration in these conditions as there is huge inter-subject variability in detecting blur or asthenopic symptoms. Thirteen subjects were advised for vision therapy as the vergence and accommodation parameters did not improve at six-month follow-ups post-surgery. Singh et al. (2015) have reported that there was an improvement in the stereo-acuity post-Lasik surgery, whereas in this study there was no significant difference in stereo-acuity before and after the surgery. The potential reason could be due to the good visual acuity and low to moderate ranges of myopia, with optimal stereo-acuity even before the surgery (Day, Powers & Faktorovich 2005).

The limitations of the study were 1) small sample size being a pilot study, 2) relatively short follow-up, 3) patient symptoms not reported, and 4) lack of a control group. The future scope of the study would be to evaluate the stability or fluctuations of the parameters over a longer duration (> six months), employ a subjective questionnaire to assess the role of vision therapy for BV anomaly after LVCS. Myopic Laser Vision Correction Surgery can precipitate or aggravate an existing non-strabismic BV anomaly. Comprehensive BV assessment and appropriate management is recommended before and after LVCS.

## CONCLUSION

The amplitude of accommodation shows significant reduction following laser refractive correction procedure in young individuals, which did not improve even after six months. So, laser refractive surgery could lead to slight reduction in accommodation for adults even before presbyopia and might precipitate or aggravate an existing non-strabismic binocular vision anomaly. Comprehensive BV assessment and appropriate management are recommended before and after LVCS.
